# Cold-Induced DHRS4 Promotes Thermogenesis via Enhanced Fatty Acid β-Oxidation in Porcine Subcutaneous Adipocytes

**DOI:** 10.3390/ani15091190

**Published:** 2025-04-22

**Authors:** Xiangfei Ma, Zijian Ye, Mengting Li, Wei Wei, Jie Chen, Lifan Zhang

**Affiliations:** College of Animal Science and Technology, Nanjing Agricultural University, Nanjing 210095, China; 2021205002@stu.njau.edu.cn (X.M.); 2022205002@stu.njau.edu.cn (Z.Y.); 2022105020@stu.njau.edu.cn (M.L.); wei-wei-4213@njau.edu.cn (W.W.); jiechen@njau.edu.cn (J.C.)

**Keywords:** cold adaptation, fatty acid β-oxidation, dehydrogenase/reductase (SDR family) member 4

## Abstract

Adipose tissue exhibits remarkable plasticity in adapting to thermal stress, yet the epigenetic mechanisms coordinating metabolic reprogramming in large mammals—particularly in livestock species lacking classical brown adipose tissue (BAT) such as swine—remain elusive. Using a porcine cold exposure model, we investigated adipose adaptation mechanisms through integrated single-cell RNA sequencing and bulk transcriptomic analyses of subcutaneous adipose tissue (subWAT). We identified a cold-induced thermogenic adipocyte subpopulation, characterized by upregulated DHRS4 expression. Mechanistically, cold exposure induced hypomethylation at the DHRS4 promoter locus, enhancing its expression to potentiate fatty acid β-oxidation, accompanied by thermogenic capacity upregulation. Our findings establish DHRS4 as an epigenetic–metabolic switch governing cold adaptation and a potential target for improving cold resistance in swine production systems.

## Simple Summary

Understanding how pigs adapt to cold environments is crucial for improving livestock welfare and productivity. Unlike humans and mice, pigs lack classical brown fat, a tissue specialized for generating heat. In this study, we explored how pigs use their fat tissue to combat cold stress. By exposing pigs to cold conditions and analyzing their fat cells, we discovered a unique subpopulation of fat cells that become active under cold temperatures. These cells increase the activity of a gene called *DHRS4*, which helps break down fats to produce heat. Cold exposure triggers changes in the chemical marks on the *DHRS4* gene (a process called hypomethylation), making it easier for the gene to function. This discovery highlights *DHRS4* as a key regulator of cold adaptation in pigs. Our findings not only deepen the understanding of how large mammals survive in harsh climates but also suggest that targeting *DHRS4* could help breed pigs with better cold resistance, ultimately reducing economic losses in livestock farming caused by cold stress.

## 1. Introduction

Adipose tissue, classically recognized as the primary energy reservoir, plays pivotal roles in maintaining metabolic homeostasis and thermoregulation [1]. Emerging evidence reveals its remarkable heterogeneity and metabolic plasticity, enabling dynamic adaptation to environmental stimuli through structural remodeling and functional metabolic reprogramming [2,3,4]. Central to this regulatory capacity are two antagonistic processes: lipogenesis and lipolysis. Lipogenesis encompasses the de novo synthesis of fatty acids (FAs) and subsequent triglyceride (TG) esterification within adipocytes, serving as a critical buffer against nutrient surplus [5,6]. Conversely, lipolysis mediates TG hydrolysis into free fatty acids (FFAs) and glycerol, mobilizing energy substrates for systemic utilization [7]. The β-oxidation pathway, a downstream process of lipolysis, subsequently catabolizes FFAs into acetyl-CoA through sequential enzymatic reactions. This multi-step degradation represents the rate-limiting phase of FA oxidation, generating substantial ATP yield while maintaining lipid homeostasis [8,9].

Adipose tissue orchestrates multilayered responses to cold exposure through its tripartite composition: energy-storing white adipose tissue (WAT), thermogenic brown adipose tissue (BAT), and the interconvertible beige adipose tissue. Cold stimulation induces WAT browning—a phenotypic transition characterized by lipid droplet multilocularization, enhanced mitochondrial biogenesis, and upregulated thermogenic gene expression (e.g., *UCP1*, *PRDM16*), especially in rodents [10,11,12]. Notably, porcine models exhibit cold-induced browning capacity despite lacking constitutive BAT. Under acute cold stress (4 °C, 10 h), subcutaneous white adipose tissue in piglets shows significant browning, with upregulation of browning marker genes *UCP1*, *PRDM16*, and *C/EBPβ*, as well as mitochondrial thermogenic genes *CIDEa*, *UCP3*, *CKMT1*, and *PM20D1* [13]. Paradoxically, chronic cold adaptation (8 °C, 15 days) suppresses both browning markers and mitochondrial biogenesis [13]. The dichotomous responses to acute versus chronic cold exposure highlight the temporal heterogeneity of WAT adaptation to thermal challenge. Lipidomic analyses combined with transcriptomic profiling of adipose tissue from cold-stressed piglets demonstrate that cold exposure induces significant alterations in the content and composition of saturated fatty acids within subcutaneous adipose tissue [14]. In addition, these analyses reveal enhanced oxidative metabolism and energy mobilization potential in SAT under cold stress [14]. These findings underscore the temporal dynamics and metabolic flexibility of adipose tissue during thermal adaptation.

DHRS4 (NADP(H)-dependent retinol dehydrogenase/reductase 4), a member of the short-chain dehydrogenase/reductase (SDR) superfamily, catalyzes sequential oxidation of retinol to retinaldehyde during all-*trans* retinoic acid (atRA) biosynthesis [15,16,17]. Beyond its canonical role in retinoid homeostasis, DHRS4 exhibits promiscuous carbonyl reductase activity toward diverse substrates, including ketosteroids and quinones [18]. Paradoxically, current research focuses predominantly on its antisense transcript *DHRS4-AS1* in carcinogenesis [19,20,21,22], while its potential metabolic functions in adipose biology remain unexplored.

Herein, we delineate the cold-adaptive reprogramming of porcine subcutaneous WAT through integrated single-cell RNA sequencing and bulk transcriptomic profiling. We identify a cold-induced thermogenic adipocyte subpopulation associated with unregulated DHRS4 expression, which coordinates enhanced fatty acid β-oxidation. Mechanistically, cold exposure attenuates CpG methylation at the DHRS4 promoter, enhancing its expression to potentiate thermogenic capacity. These findings elucidate an epigenetic–metabolic crosstalk in cold adaptation and propose DHRS4 as a translational target for improving porcine cold resistance, with implications for sustainable livestock production under cold stress.

## 2. Materials and Methods

### 2.1. Animals

A cold stimulation test was conducted on 6–8-week-old male Erhualian piglets with similar body weights. Littermates were divided into a room temperature group (28 ± 0.5 °C) and a cold stimulation group (11 ± 0.5 °C), with 3 piglets per group. All animals were individually housed in cages with ad libitum access to food. The cold exposure trial lasted for one month. All processes involving animal experiments in this study were reviewed and approved by the Institutional Animal Care and Use Committee of Nanjing Agricultural University under NJAU. No20220317047.

### 2.2. Data Analysis of snRNA-Seq

Subcutaneous adipose tissue samples from the dorsal region was pooled within groups for snRNA-seq. Sequencing data are deposited at the China National Center for Bioinformation/Beijing Institute of Genomics, Chinese Academy of Sciences, under accession numbers GSA: CRA020162 and GSA: CRA020109. Data analysis was performed using the Omicsmart platform provided by Genedenovo Biotechnology.

### 2.3. Isolation of SVF and Differentiation of Preadipocytes

Subcutaneous adipose tissue was harvested from the dorsal region of freshly slaughtered piglets. The tissue was finely minced using sterile scissors and subsequently digested with 0.1% Type I collagenase (Biosharp, BS163, Hefei, Anhui, China) in a shaking water bath at 37 °C for 1 h. Following the termination of digestion by adding an equal volume of complete culture medium, the digestate was filtered through a 100 μm nylon mesh. The filtrate was centrifuged at 1000 rpm for 5 min at room temperature. The resulting cell pellet was resuspended in complete culture medium and seeded into T25 culture flasks, followed by incubation at 37 °C in a humidified atmosphere of 5% CO_2_.

Once the stromal vascular fraction (SVF) cells or ISP4# cells (an immortalized porcine adipocyte cell line) [23] reached confluence and were contact-inhibited for 2 days, a differentiation induction medium (containing 0.5 mM 3-isobutyl-1-methylxanthine (Sangon, A606630, Shanghai, China), 125 μM indomethacin (Makclin, I811784, Shanghai, China), 1 μM dexamethasone (Sangon, A601187, Shanghai, China), 1 μg/mL insulin (Novolin R, Tianjin, China), 1 nM T3 (Makclin, T819947, Shanghai, China), and 1 μM rosiglitazone (Makclin, R832516, Shanghai, China) was added to induce differentiation for 4 days. Subsequently, the medium was replaced with maintenance medium (containing 1 μg/mL insulin, 1 nM T3 and 1 μM) for an additional 4 days.

### 2.4. Oil Red O Staining

The differentiated SVF cells or ISP4# cell line were washed three times with phosphate-buffered saline (PBS, Servicebio, G4202, Wuhan, China) and then fixed with 4% paraformaldehyde for 30 min. After fixation, the cells were washed three times with PBS. A freshly prepared Oil Red O working solution (Oil Red O/distilled water = 3:2) was added to cells, which were then incubated at room temperature for 30 min. Excess Oil Red O staining solution was removed by washing with PBS. Images were captured under a microscope, and then an equal volume of isopropanol was added to each well to extract the stain. The absorbance was measured at 510 nm using a microplate reader for quantitative analysis.

### 2.5. Construction of DHRS4 Cell Line

To construct the lentiviral overexpression plasmid, the coding sequence of DHRS4 (NM_214019) was cloned into the SmaI site of the pLVX-V5-IRES-neo vector using the 2 × Hieff Clone^®^ Universal II Enzyme Premix (YEASEN, 10923ES, Shanghai, China) for homologous recombination. The primers used for DHRS4 cloning are listed in Supplementary Table S1. To generate lentiviral particles, the lentiviral construct was co-transfected with pM2D.G and psPAX2 into HEK293T cells. The lentivirus-containing medium was harvested, filtered, and concentrated at 48 and 72 h post-transfection. The immortalized preadipocyte cell line ISP4# was then incubated with the lentivirus (Servicebio, G1803, Wuhan, China) premixed with polybrene (10 μg/mL, Solarbio, H8761, Beijing, China) for 72 h. Positive cells were selected by adding 800 μg/mL G418 antibiotic (Beyotime, ST081, Shanghai, China) and subsequently expanded to meet experimental requirements.

### 2.6. Overexpression of DHRS4 in SVF Cells

The control vector pLVX-V5-IRES-neo and the DHRS4 overexpression vector pLVX-V5-DHRS4-IRES-neo were transfected into SVF cells according to the Lipofectamine 3000 transfection protocol (Thermo Fisher, Waltham, MA, USA). After the cells reached contact inhibition, they were induced to differentiate. Subsequently, the cells were collected for qPCR or Western blot analysis.

### 2.7. RNA Isolation and RT-qPCR

Total RNA was isolated from cells or tissues using TRIzol reagent (Accurate Biology, AG21101, Changsha, Hunan, China). For reverse transcription, 1 µg of RNA was used to synthesize cDNA according to the manufacturer’s instructions (Accurate Biology, AG11706, Hunan, China). RT-qPCR was performed on an ABI (Applied Biosystems, Waltham, MA, USA) instrument using SYBR qPCR Master Mix (YEASEN, 11184ES, Shanghai, China). Relative mRNA levels were calculated using the 2^(−ΔΔCT)^ method, with RPLP0 serving as the internal reference gene for normalization. The sequences of the quantitative primers used are listed in Supplementary Table S1.

### 2.8. Western Blotting

Proteins were extracted from tissues or cells using RIPA buffer (NCM Biotech, WB3100, Suzhou, China) supplemented with protease inhibitors (Beyotime, P1050, Shanghai, China). An enhanced chemiluminescence Western-blotting substrate (Biosharp, BL520B, Anhui, China) was used for visualization of the results. The following antibodies were employed to detect the target proteins: anti-Tubulin (Proteintech, 11224-1-AP, Wuhan, China), anti-DHRS4 (Proteintech, 15279-1-AP, Wuhan, China), anti-UCP3 (Abclonal, A23285, Wuhan, China), anti-PPARG (Proteintech, 16643-1-AP, Wuhan, China), anti-HSL, (Proteintech, 17333-1-AP, Wuhan, China), and anti-CPT1A (Proteintech, 15184-1-AP, Wuhan, China).

### 2.9. Analysis of DNA Methylation Levels in the Promoter Region of DHRS4

DNA was extracted from the subcutaneous adipose tissue of pigs in both the RT group and the Cold group. The extracted DNA was subsequently subjected to bisulfite conversion using a commercial kit (TIANGEN, DP215-02, Beijing, China). Methylation islands within the promoter region of DHRS4 were predicted using the online software MethPrimer (https://www.methprimer.com/), and methylation-specific primers were subsequently designed based on these predictions. Subsequently, the predicted methylation islands within the DHRS4 promoter region were cloned, and the resulting fragments were ligated into a vector for further analysis. Ten single clones from each group (RT and Cold) were selected for sequencing to quantify the DNA methylation levels within the promoter region of DHRS4.

### 2.10. Determination of Free Fatty Acids

Collect the cell culture medium from the control group and the DHRS4-overexpressing group in ISP4# cells. Prepare the free fatty acid detection working solution according to the manufacturer’s instructions (Beyotime, S0215S, Shanghai, China), and subsequently measure the samples at a wavelength of 570 nm.

### 2.11. Statistical Analysis

Statistical analysis was performed using Prism 8.0 (GraphPad). Analysis between two groups was performed using a two-tailed unpaired Student’s *t*-test. Sample size has been indicated in each figure legend. The values are expressed as mean ± SEM. *p*-values are indicated as * *p * ≤  0.05; ** *p*  ≤  0.01; *** *p*  ≤  0.001.

## 3. Results

### 3.1. Subclusters of Adipocytes at Single-Cell Resolution

**Figure 1:**
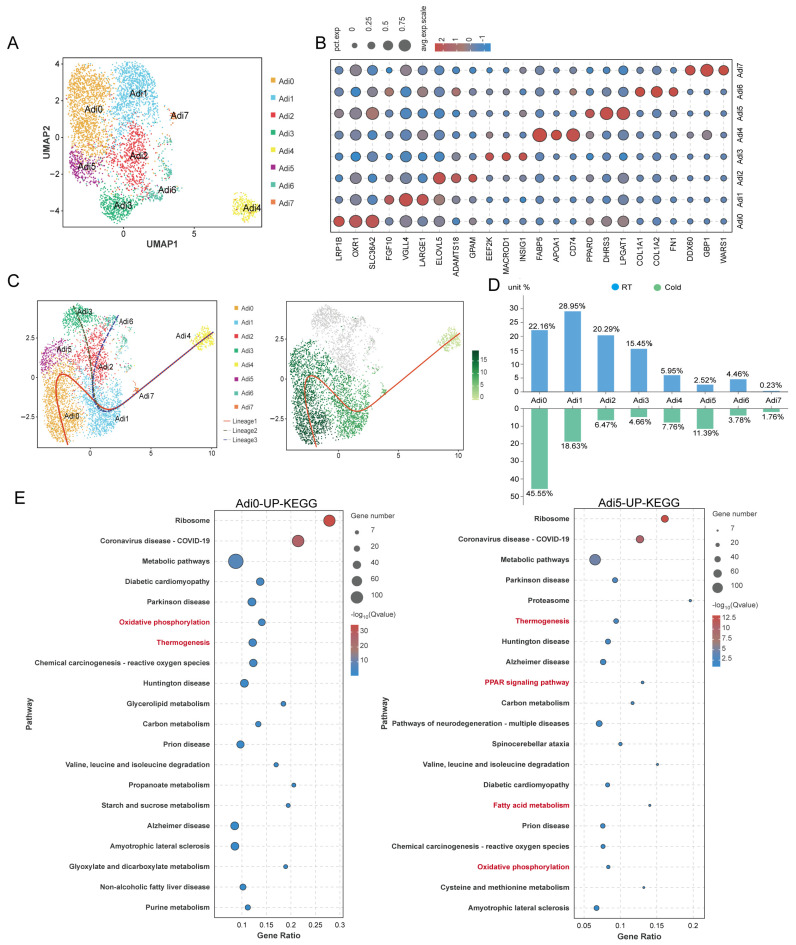
(**A**) UMAP of adipocytes subpopulations. (**B**) Bubble chart showing scaled average expression of adipocytes subpopulations-enriched marker genes. (**C**) The trajectory inference and state information of adipocytes subpopulations. Each dot represents a single cell. (**D**) The fraction of each adipocyte’s subpopulation in dorsal subWAT from the RT group and the Cold group. (**E**) The top 20 enrichment pathways revealed by KEGG enrichment analysis of upregulated differentially expressed genes in Adi0, Adi5 subpopulations. The red font color indicates metabolic pathways related to thermogenesis.

To characterize the heterogeneity of adipocytes in the subcutaneous adipose tissue of piglets, we conducted re-clustering analysis, identifying eight distinct adipocyte subpopulations (Figure 1A). The highly expressed genes in each cluster are presented in Figure 1B. Pseudotemporal differentiation trajectory analysis using Slingshot2 revealed three distinct differentiation trajectories among the adipocyte subpopulations. Cells in differentiation trajectory 1 differentiated into a population exhibiting robust antioxidant capacity and enhanced energy metabolism, marked by the high expression of genes, including LRP1B, OXR1, SLC36A2, PPARD, DHRS3, and LPGAT1. The cell subpopulation Adi3 in differentiation trajectory 2 differentiated into an insulin-sensitive cell population, characterized by high expression of genes, including EEF2K, MACROD1, and INSIG1. The cell subpopulation Adi6 in differentiation trajectory 3 predominantly differentiated into fibrotic adipocytes, exhibiting high expression of COL1A1, COL1A2, and FN1 (Figure 1B,C). Following cold exposure in piglets, the proportion of cells in the Adi0 and Adi5 subpopulations, which exhibit robust antioxidant capacity and enhanced energy metabolism, significantly increased (Figure 1D). We performed KEGG enrichment analysis on the upregulated differentially expressed genes in adipocyte subpopulations Adi0 and Adi5 following cold exposure. The results revealed significant enrichment in pathways associated with thermogenesis, oxidative phosphorylation, and PPAR signaling (Figure 1E), indicating that the Adi0 and Adi5 subpopulations are thermogenesis-related adipocytes.

### 3.2. Upregulation of DHRS4 Expression Was Observed in the Cold-Exposed Group

**Figure 2:**
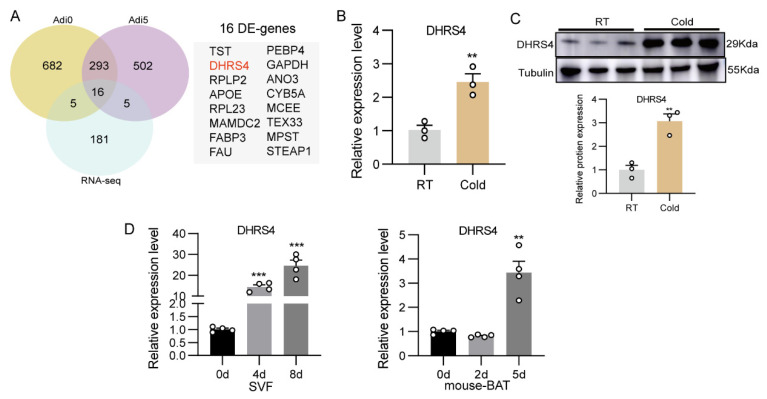
(**A**) I. Venn diagram showing the overlapping genes that DEGs of Adi0 and Adi5 subpopulations significantly upregulated and RNA-seq of dorsal subWAT. (**B**,**C**) qPCR and Western Blotanalysis of DHRS4 expression in dorsal subWAT at the RT group and the Cold group. (**D**) Expression patterns of DHRS4 during the differentiation of SVF Cells and mouse Brown Adipocytes. ** *p*  ≤  0.01; *** *p*  ≤  0.001.

To identify candidate genes associated with adaptive thermogenesis, we performed an integrated analysis of differentially expressed genes (DEGs) in the adipocyte subpopulations Adi0 and Adi5, combined with DEGs derived from RNA-seq data of dorsal subcutaneous adipose tissue in cold-exposed piglets. A total of 16 DEGs were identified, among which DHRS4 was one of the significantly differentially expressed genes (Figure 2A). qPCR and Western blot analyses demonstrated that DHRS4 was significantly upregulated in the dorsal subcutaneous adipose tissue of cold-exposed piglets (Figure 2B,C). Furthermore, the expression pattern of DHRS4 during porcine adipocyte differentiation closely resembled that observed in murine brown adipocyte differentiation, showing a marked upregulation during the late differentiation stage (Figure 2D). These findings show that DHRS4 may play a critical role in the differentiation of thermogenic adipocytes.

### 3.3. DHRS4 Enhances Thermogenesis in ISP4# Cells by Upregulating Fatty Acid β-Oxidation

**Figure 3:**
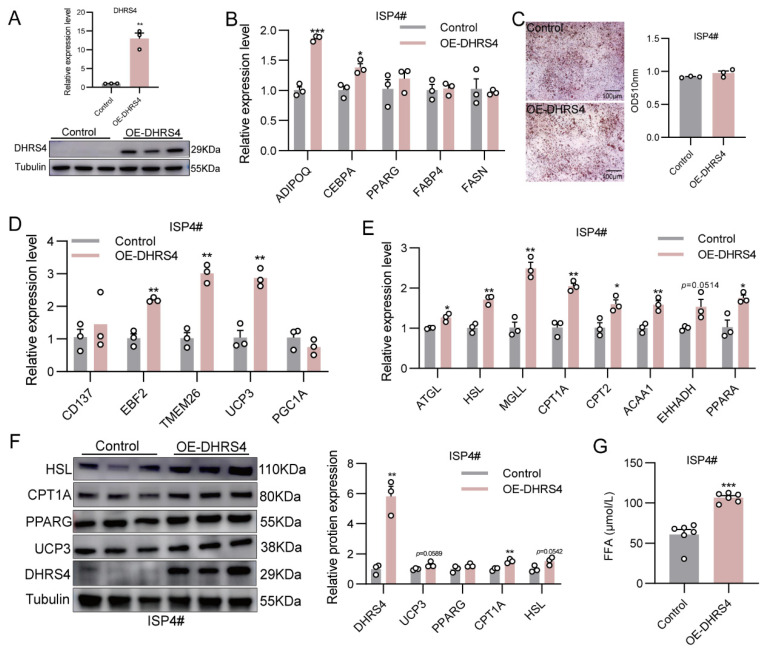
(**A**) Detection of DHRS4 overexpression efficiency in ISP4# cells. (**B**) qPCR analysis of adipogenic differentiation marker genes following DHRS4 overexpression in ISP4# cells at differentiation day 8. (**C**) Oil Red O staining of ISP4# cells following DHRS4 overexpression at differentiation day 8. (**D**,**E**) qPCR analysis of genes related to thermogenesis, lipolysis, and fatty acid β-oxidation following DHRS4 overexpression in ISP4# cells at differentiation day 8. (**F**) Western blot analysis of HSL, CPT1A, PPARG, UCP3, and DHRS4 expression levels in ISP4# cells overexpressing DHRS4 at differentiation day 8. (**G**) Free fatty acid (FFA) content in culture medium of ISP4# cells overexpressing DHRS4 at differentiation day 8. * *p * ≤  0.05; ** *p*  ≤  0.01; *** *p*  ≤  0.001.

To investigate the function of DHRS4, we established a stable ISP4# cell line overexpressing DHRS4. The successful overexpression of DHRS4 was confirmed by qPCR and Western blot analysis (Figure 3A). Overexpression of DHRS4 significantly upregulated the expression of adipogenic markers ADIPOQ and CEBPA (Figure 3B). However, Oil Red O staining revealed that DHRS4 overexpression did not significantly alter lipid droplet accumulation in ISP4# cells (Figure 3C). To further explore the role of DHRS4 in thermogenesis, we analyzed the expression of thermogenesis-related marker genes using qPCR. The results demonstrated that DHRS4 overexpression significantly upregulated the expression of EBF2, TMEM26, and UCP3 in ISP4# cells (Figure 3D). Given the close association between fatty acid β-oxidation and adaptive thermogenesis, we examined the expression of key genes involved in fatty acid β-oxidation. Our findings indicated that DHRS4 overexpression significantly enhanced the expression of lipolysis-related genes (ATGL, HSL, MGLL) and fatty acid β-oxidation-related genes (CPT1A, CPT2, ACAA1, EHHADH, PPARA) in ISP4# cells (Figure 3E). Western blot analysis further revealed that DHRS4 overexpression significantly increased the protein level of CPT1A, while the levels of UCP3 and HSL exhibited an upward trend without reaching statistical significance. The protein level of PPARG remained unchanged (Figure 3F). Additionally, the FFA content in the culture medium of ISP4# cells were significantly elevated following DHRS4 overexpression (Figure 3G).

### 3.4. DHRS4 Promotes Thermogenesis in SVF via Fatty Acid β-Oxidation

**Figure 4:**
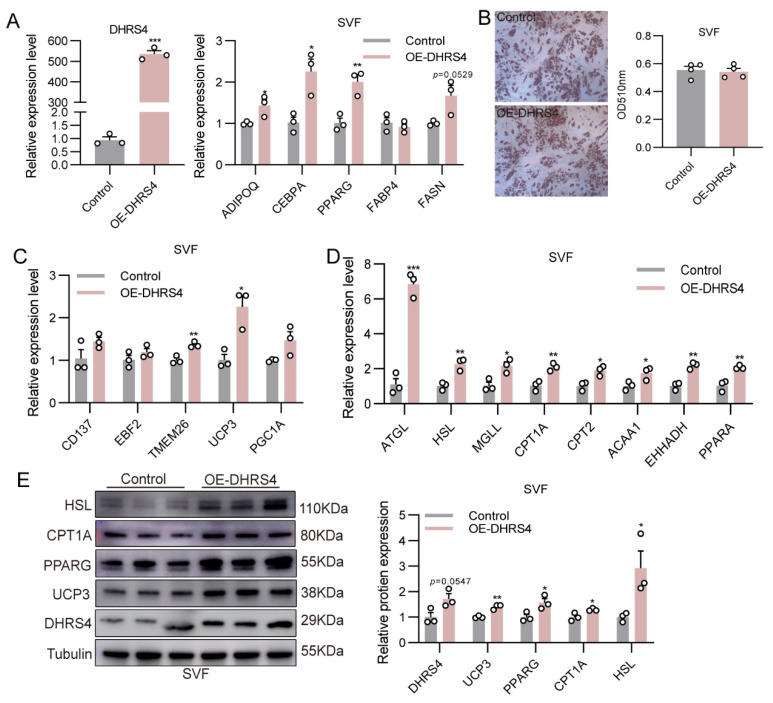
(**A**) Detection of DHRS4 overexpression efficiency and qPCR analysis of adipogenic differentiation marker genes following DHRS4 overexpression in SVF cells at differentiation day 8. (**B**) Oil Red O Staining of SVF cells following DHRS4 overexpression at differentiation day 8. (**C**,**D**) qPCR analysis of genes related to thermogenesis, lipolysis, and fatty acid β-oxidation following DHRS4 overexpression in SVF cells at differentiation day 8. (**E**) Western blot analysis of HSL, CPT1A, PPARG, UCP3, and DHRS4 expression levels in SVF cells overexpressing DHRS4 at differentiation day 8. * *p * ≤  0.05; ** *p*  ≤  0.01; *** *p*  ≤  0.001.

To validate the thermogenic function of DHRS4, we overexpressed DHRS4 in porcine primary SVF cells and examined the expression of genes associated with adipocyte differentiation, thermogenesis, lipolysis, and fatty acid β-oxidation. Compared to the control group, DHRS4 overexpression significantly upregulated the expression of adipogenic markers (ADIPOQ, CEBPA, and PPARG) (Figure 4A). However, the quantification analysis of Oil Red O staining revealed no significant differences in lipid droplet accumulation between the groups (Figure 4B). Notably, the expression of beige adipocyte marker genes (TMEM26 and UCP3) was significantly elevated in the DHRS4 overexpression group (Figure 4C). Moreover, DHRS4 overexpression enhanced the expression of lipolysis-related genes (ATGL, HSL, MGLL) and fatty acid β-oxidation-related genes (CPT1A, CPT2, ACAA1, EHHADH, PPARA) in SVF cells (Figure 4D). Consistent with these findings, the protein levels of CPT1A, HSL, PPARG, and UCP3 were also significantly increased in the DHRS4 overexpression group (Figure 4E).

### 3.5. Cold Exposure Activates the Methylation of the DHRS4 Promoter Region

**Figure 5:**
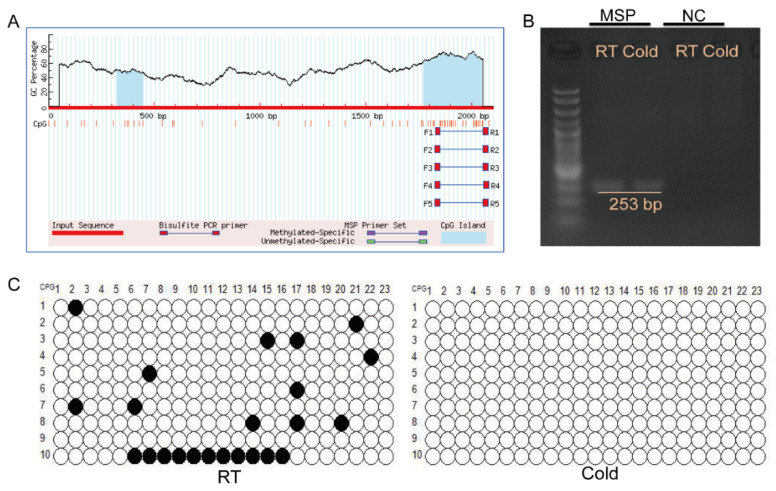
(**A**) Prediction of CpG Islands in the DHRS4 promoter using MethPrimer software (https://www.methprimer.com/). (**B**) Amplification of DHRS4 CpG sites using methylation-specific primers in dorsal subWAT from RT and Cold groups. (**C**) Methylation patterns of CpG islands in the DHRS4 promoter region in dorsal subWAT from RT and Cold groups. Black circles represent methylated sites, while white circles denote unmethylated sites.

To elucidate the mechanism underlying DHRS4 activation under cold exposure, we predicted potential DNA methylation sites within the 2000 bp upstream region of the DHRS4 transcription start site. Our analysis identified a 273 bp CpG island located within the promoter region of DHRS4 (Figure 5A). Methylation-specific primers (MSP) and control primers were designed to amplify the CpG island in bisulfite-converted genomic DNA. The results demonstrated that the MSP primers successfully amplified the target fragment, whereas no amplification was detected with the control primers, thereby confirming the efficiency of bisulfite conversion (Figure 5B). Methylation levels were quantified using the MSR Calculate tool, revealing that the 23 CpG sites within the DHRS4 promoter region displayed significantly higher methylation levels in the room temperature group (10%) compared to the cold exposure group (0%) (Figure 5C).

## 4. Discussion

Our integrative multi-omics approach delineates a previously unrecognized epigenetic–metabolic axis through which cold stress primes porcine subcutaneous WAT for thermogenic adaptation. By coupling single-cell resolution with bulk transcriptomic profiling, we identified DHRS4 as a cold-responsive epigenetic rheostat coordinating β-oxidation enhancement. This finding bridges the critical knowledge gap between environmental sensing and adipose remodeling in large mammals. Our multi-omics dissection highlights the innovation of our approach in elucidating the complex regulatory networks underlying adipose tissue metabolic plasticity.

The hypomethylation-mediated DHRS4 activation observed at chr7: 75, 257, 594-75, 255, 495 (GCF_000003025.6, Sus scrofa 11.1) in our study echoes evolutionarily conserved epigenetic mechanisms in murine adaptive thermogenesis. Preconception cold exposure reduces CpG methylation in paternal sperm (89.5% to 87.5%), conferring offspring with enhanced brown adipogenesis, neurogenesis, and cold tolerance through transgenerational epigenetic inheritance [24]. A recent integrated analysis of DNA methylomes and transcriptomes from BAT of male C57BL/6J mice exposed to thermoneutral (28.8 °C), mild cold (22 °C), or severe cold (8 °C) conditions revealed temperature-dependent hypomethylation inversely correlated with gene expression thermogenic gene expression [25]. Notably, cold exposure markedly reduces methylation of the Vaspin gene (encoding a serine protease inhibitor with anti-diabetic and anti-obesity properties) in BAT [26], collectively supporting epigenetic regulation as an evolutionarily conserved strategy for adipose thermogenesis.

Retinol metabolism exerts pleiotropic effects on adipogenesis through isoform-specific mechanisms. Retinol-binding proteins (RBPs), essential carriers for retinol transport, act as metabolic modulators. Overexpression of RBP7 in 3T3-L1 preadipocytes promotes differentiation and triglyceride accumulation via upregulation of PPARγ, FABP4, C/EBPα, and AdipoQ, whereas RBP7 deficiency inhibits adipogenesis—a phenotype rescued by retinoic acid (RA) [27]. Conversely, ectopic RBP4 expression reduces adipocyte size and inguinal fat deposition in mice [28], while RBP4 suppresses porcine preadipocyte differentiation by attenuating insulin signaling [29]. Retinol dehydrogenase 10 (RDH10), the rate-limiting enzyme in RA biosynthesis, finetunes lipid metabolism through RA biosynthesis, as evidenced by RDH10 deficiency elevates CD36 expression (a key free fatty acid receptor) via RA depletion [30]. Building on this regulatory framework, our study demonstrates that DHRS4 enhances adipocyte β-oxidation to potentiate thermogenesis. Given its dual roles in retinoid metabolism and carbonyl reduction, we propose two non-exclusive mechanisms: (1) DHRS4-driven RA production may activate PPARα/RXR heterodimers, thereby upregulating β-oxidation genes (CPT1a, CPT2, ACAA1; Figure 3E), which in turn promotes the expression of thermogenic genes (UCP3, EBF2, TMEM26; Figure 3D); (2) NADPH flux during retinol reduction by DHRS4 may counteract cold-induced oxidative stress. While establishing the DHRS4-β-oxidation axis, critical questions emerge from these findings: (1) How do its catalytic and putative non-enzymatic functions coordinate?? (2) Is this mechanism depot-specific (subcutaneous vs. visceral adipose tissue)? These findings advance our understanding of adipose thermoregulation, yet further investigations are warranted to dissect the multifunctional roles of DHRS4.

Beyond porcine thermoregulation, our work illuminates an evolutionary paradigm where retrograde signals from environmental stressors (e.g., cold) converge on epigenetic–metabolic interfaces to drive adipose adaptation—a mechanism potentially relevant to human metabolic disorders exacerbated by chronic “thermal stress” in modern lifestyles. This conceptual framework suggests that understanding the interplay between environmental cues and epigenetic regulation could reveal novel therapeutic targets for cold counteraction.

While this study provides novel insights into porcine adipose adaptation, certain limitations should be noted. First, the sample size (*n* = 3 per group), may reduce statistical power for low-effect-size phenotypes. Second, while multi-omics convergence compensated for sample limitations in high-resolution analyses, techniques like Western blotting and qPCR inherently require larger cohorts for subtle effects. Future studies should employ longitudinal designs with staggered sampling to meet higher-precision statistical demands. Crucially, our findings lay the groundwork for validating identified biomarkers (e.g., *DHRS4*) in commercial pig populations under real-world cold stress.

## 5. Conclusions

In this study, we explored the adaptive mechanisms of adipose tissue in response to cold stress using a porcine model, focusing on the epigenetic regulation of metabolic reprogramming in large mammals lacking classical BAT. Through integrated single-nucleus RNA sequencing and transcriptomic analyses of subWAT, we identified a thermogenic adipocyte subpopulation that emerges under cold stress, characterized by the upregulation of DHRS4 expression. Mechanistically, cold exposure induced hypomethylation at the DHRS4 promoter locus, enhancing its transcriptional activity and promoting fatty acid β-oxidation, alongside increased thermogenic capacity. These findings establish DHRS4 as a critical epigenetic–metabolic switch that governs cold adaptation in swine. Our results not only advance the understanding of adipose tissue plasticity in large mammals but also highlight DHRS4 as a potential target for improving cold resistance in swine production systems, offering valuable insights for mitigating the adverse effects of cold stress on livestock health and productivity.

## Data Availability

Data are contained within the article.

## Supplementary Materials

The following supporting information can be downloaded at https://www.mdpi.com/article/10.3390/ani15091190/s1: Table S1: Primers for RT-qPCR.
