# Supplementary material for: Cold-Induced DHRS4 Promotes Thermogenesis via Enhanced Fatty Acid β-Oxidation in Porcine Subcutaneous Adipocytes
Source: Animals (Basel). 2025 Apr 22;15(9):1190. doi: 10.3390/ani15091190 (PMC12071078; doi:10.3390/ani15091190)
Supplement: Supplementary file 1 [file animals-15-01190-s001.zip › animals-3520977-supplementary.pdf]

Supplementary Table S1. Primers for RT-qPCR.

**Primer**	**Sequence （5’ to 3’）**
DHRS4-CDS-F	CGGTACCGCGGGCCCgccaccatgcgggcggcgggg
DHRS4-CDS-R	ggcttaccGGcgATCCCgaggcgggatgcggtccctc
Pig RPLP0-F	GCACCTGGAAAACAACCCAG
Pig RPLP0-R	GGCAGCTGGCACCTTATTG
Pig DHRS4-F	GCCGTCAACCCATTCTTTGG
Pig DHRS4-R	GCACCACTGCCTTTGTCATC
Pig ATGL-F	TCCTCCCTAGTGGCCAACG
Pig ATGL-R	TCGACACCTCGATGATGCTG
Pig HSL-F	ACACAAGGGCTGCTTCTACG
Pig HSL-R	CTGAGGCCTGTTTCATTGCG
Pig UCP3-F	AGTGGATGTGGTGAAGACCC
Pig UCP3-R	TTCCCAAGCGCAAAAAGGAAG
Pig PGC1A-F	GCAGTTCTCACAGAGACGCT
Pig PGC1A-R	TAGAGACGGCTCTTCTGCCT
Pig CD137-F	TCTCCAGGTCACACTTCCCA
Pig CD137-R	GCTGGCTTCAGAAACGGTTG
Pig TMEM26-F	GTGGGATCACTCGGGATCAA
Pig TMEM26-R	TAAGGATGGCGTAGACCAGG
Pig Adipoq-F	AGCGCCTATGTCTACCGTTC
Pig Adipoq-R	AGCGAATGGGCATGTTAGGG
Pig FABP4-F	CCCTGGTACAGGTGCAGAAG
Pig FABP4-R	TGGTAGCCGTGACACCTTTC

Pig FASN-F	CCCGAATCTGCACTACCACA
Pig FASN-R	AGCCGAAGGAGTTTATGCCC
Pig PPARG-F	CAGGTCCACAGAGCTGATCC
Pig PPARG-R	AGAAAGCGATGCCTTCGACA
Pig CEBPA-F	CTCACCGCTCCGATTCCTAC
Pig CEBPA-R	TCCTTCTATTGCGGGGGAGA
Pig EBF2-F	AACAGTTCTGCAAAGGAGCG
Pig EBF2-R	TCCAGGATGTCTCGGGATGA
Pig MGLL-F	CCCTGGTCTTTGTGTCGCA
Pig MGLL-R	ATCCTCTCTCCCTCGCTCTG
Pig CPT1A-F	TGGTGTCCAAATACCTCGCC
Pig CPT1A -R	CCTCCGCTCGACACATACTC
Pig CPT2-F	ATTCCACTCGACTCCCCAGA
Pig CPT2-R	CGCTCACAATCTTCCCGTCT
Pig ACAA1-F	TTCAAGGACACCACCCCTGAC
Pig ACAA1-R	GAAGCACATTTCCCACGCA
Pig EHHADH-F	TCTGTTGGCATTCTCGGCTT
Pig EHHADH-R	CACGGGGATTTTGGCCTTTG
Pig PPARA-F	TTTCCACAAGTGCCTCTCGG
Pig PPARA-R	CGTCTTCTCGGCCATACACA
NC-CpG-F	ACTGGGAGACTGCAAGACATCA
NC- CpG-R	GGGAAGTTGACAAAATAGAACCAGC
MSP-CpG-F	GTTTAAACTTAAGCTATGTGGATTGGGAGATTGTAAGATATTA
MSP- CpG-R	GATATCTGCAGAATTAAAAAATTAACAAAATAAAACCAAC
